# Refractory ascites: unveiling POEMS syndrome as the underlying cause: a case report and literature review

**DOI:** 10.3389/fmed.2025.1704902

**Published:** 2025-12-01

**Authors:** Shuang Liu, Yin He, Jiamin Qin, Li Wang, Xinhui He, Yingyue Zhou, Qin He, Kaixiao Kun, Liming Wen

**Affiliations:** 1 Clinical Medicine College, Southwest Medical University, Luzhou, Sichuan, China; 2 Department of Gastroenterology, Mianyang 404 Hospital, Mianyang, Sichuan, China; 3 School of Medicine, Southwest University of Science and Technology, Mianyang, Sichuan, China

**Keywords:** POEMS syndrome, refractory ascites, diagnosis, case report, review

## Abstract

Ascites is a common clinical manifestation usually caused by portal hypertension, hypoalbuminemia, or malignant tumors; POEMS syndrome, a rare cause of ascites, can also lead to refractory ascites. However, due to its low incidence and complex clinical manifestations, this syndrome is prone to being missed or misdiagnosed, resulting in delayed treatment. This article reports an elderly male patient with refractory peritoneal effusion who had multiple unsuccessful treatments. After admission to our hospital’s Department of Gastroenterology, his symptoms persisted despite diuresis and paracentesis. Further inquiry showed he had experienced 2 years of recurrent pruritus, lower limb edema, and occasional numbness. Tests revealed IgA-*λ* type M proteinemia and elevated VEGF, while imaging showed osteosclerotic lesions and splenomegaly. Finally, He was diagnosed with POEMS syndrome via bone marrow biopsy. After treatment with bortezomib, dexamethasone, and lenalidomide, his symptoms improved significantly. POEMS syndrome, a rare cause of ascites, is frequently missed or misdiagnosed; thus, clinicians should maintain high vigilance. In patients with unexplained refractory ascites and symptoms like neuropathy, organ enlargement, and endocrine disorders, screening for VEGF levels and M protein should be performed to facilitate early identification of POEMS syndrome.

## Introduction

Ascites refers to the abnormal accumulation of fluid in the abdominal cavity, Common causes of ascites include cirrhotic portal hypertension, which accounts for the majority of cases, malignant tumors, heart failure, spontaneous bacterial peritonitis, and tuberculous peritonitis (1, 2). POEMS syndrome is a rare cause of ascites, often presenting with polyneuropathy, organomegaly, endocrine abnormalities, monoclonal gammopathy, and skin lesions. Its pathogenesis is significantly unique, which easily leads to missed diagnosis or misdiagnosis (3–5). We report a case of POEMS syndrome in an elderly patient with refractory peritoneal effusion as the main manifestation.

## Case description

**Figure 1:**
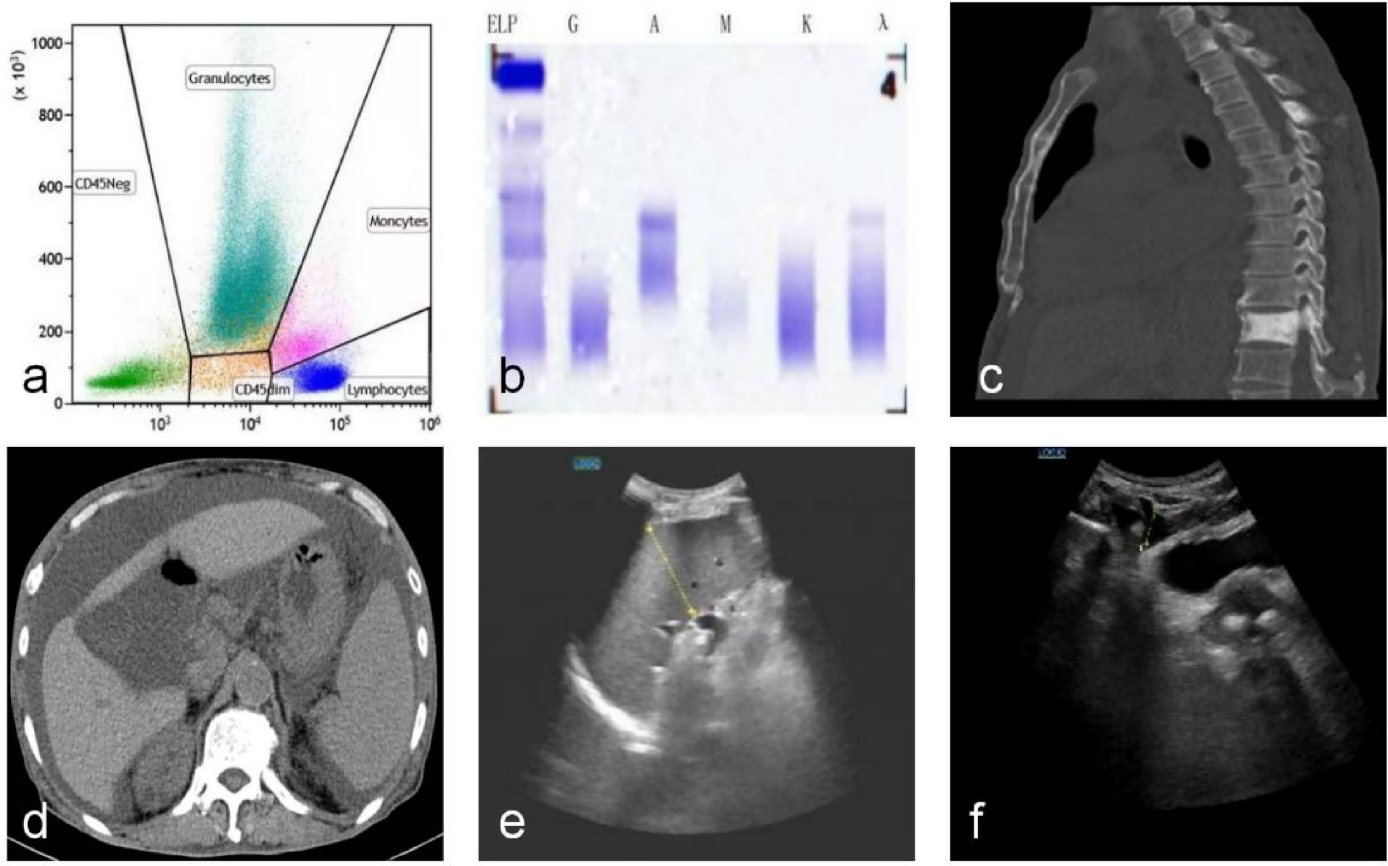
The examination results of the patient before and after treatment are as follows. **(a)** FCM detected approximately 0.11% of monoclonal plasma cells. **(b)** Analysis of serum immunofixation electrophoresis (IF) typing results: The type of monoclonal immunoglobulin is IgA-*λ*. **(c)** Pre-Treatment Thoracoabdominal CT: Patchy Hyperdense Shadows in the Cervical Spine and Multiple Thoracolumbar Vertebrae. **(d)** Pre-Treatment Abdominal CT: Large Amount of Fluid Accumulation in the Pelvic and Abdominal Cavities; Splenomegaly. **(e)** Post-Treatment Abdominal Ultrasound: Mild Splenomegaly. **(f)** Post-Treatment Ascites Ultrasound Examination: Fluid Accumulation in the Intestinal Spaces of the Lower Abdomen.

The patient was a 70-year-old male admitted to the hospital with recurrent generalized pruritus and edema accompanied by numbness in both lower limbs for 2 years. He also experienced abdominal distension for more than 20 days. Two years ago, the patient developed generalized pruritus without obvious inducement, which was accompanied by edema and occasional numbness and discomfort in both lower limbs. The patient had sought medical treatment in multiple other hospitals successively. Routine examinations of blood, ascites, and abdominal CT considered “ascites of unknown cause.” Despite symptomatic treatments such as diuresis and detumescence, the symptoms persisted. Six months ago, the patient underwent a PET/CT examination at another hospital because of bone pain. The scan revealed high uptake in the 11th thoracic vertebra and focal osteoblastic changes in multiple thoracic and lumbar vertebrae. More than 20 days before admission, the patient developed abdominal distension without obvious inducement, accompanied by progressive weight loss and intolerable skin pruritus, No specific skin changes such as hirsutism, hair loss, or skin thickening.so he went to the Department of Gastroenterology, Mianyang 404 Hospital for treatment. On physical examination after admission, the skin and mucous membranes appeared normal. No enlargement of superficial lymph nodes was palpable throughout the body. Dullness was noted on percussion of the dorsal segment of the left lung. The abdomen was distended, but the liver and spleen were not palpable below the costal margin. Shifting dullness was positive. No edema was observed in both lower limbs. Auxiliary examinations: Complete Blood Count (CBC): White Blood Cell (WBC) Count: 115 × 10^9^/L (Reference Range: 125–350 × 10^9^/L); Albumin: 35.7 g/L (Reference Range: 40–55 g/L); Thyroid function: Thyroid-stimulating hormone: 5.96 uIU/mL (Reference Range: 0.27–4.2 uIU/mL); Parathyroid hormone: 68.35 pg./mL (Reference Range: 15–65 pg./mL); Testosterone: 0.844 ng/mL (Reference Range: 1.93–7.40 ng/mL); Renal function: Creatinine: 185 μmol/L (Reference Range: 57–111 μmol/L). Routine examination of pleural and ascitic fluid: Color: pale yellow; Clarity: clear; Rivalta test: weakly positive (±); Total cell count: 918 × 10^6^/L. Biochemistry of pleural and ascitic fluid: Lactate dehydrogenase: 65 U/L; Adenosine deaminase: 3.8 U/L; Total protein: 37.7 g/L. Molecular and cytopathological analysis of ascitic fluid has found no tumor cells or *Mycobacterium tuberculosis*. Abdominal ultrasound showed massive peritoneal and pelvic effusion. Chest and abdominal CT showed nodular and patchy increased density shadows in the cervical spine, multiple thoracic and lumbar vertebrae with some accessories, the 4th left anterior rib, and the left superior pubic ramus (Figure 1c); splenomegaly; and massive pelvic and abdominal effusion (Figure 1d). Echocardiography showed a small amount of pericardial effusion. Thoracic vertebra CT showed: localized increased bone density in the C6-L1 vertebrae and some accessories, with small high-density nodules in some areas, predominantly in the T11 vertebra. The patient’s symptoms showed no significant improvement after treatments such as diuresis and abdominal puncture with catheterization for ascitic fluid drainage.

Based on the patient’s clinical manifestations and his status as an elderly male, further relevant examinations were completed. The main symptoms included refractory pleural and ascitic fluid, numbness in both lower limbs with occasional edema, renal dysfunction, mild endocrine abnormalities, and typical bone changes. The results showed the following: vascular endothelial growth factor (VEGF) was 209.88 pg./mL (Reference Range: < 160.00 pg./mL); bone marrow aspiration showed active granulocyte/erythrocyte hyperplasia, active megakaryocyte hyperplasia, and normal platelet count. Bone marrow biopsy indicated a slight increase in bone marrow plasma cells (accounting for approximately 3–5%). The results of bone marrow flow cytometry immunofluorescence analysis showed that flow cytometry (FCM) detected approximately 0.11% of monoclonal plasma cells (Figure 1a). Serum immunofixation electrophoresis revealed the monoclonal immunoglobulin type as IgA-*λ* (Figure 1b). The abnormal immunoglobulinemia report confirmed the M protein type as IgA-λ. The electromyography report showed that motor nerve conduction velocity (MCV) of both common peroneal nerves was slightly decreased, and sensory nerve conduction velocity (SCV) of both superficial peroneal nerves and both sural nerves was slightly decreased. Based on the above examination results and consultation opinions from the Department of Hematology, POEMS syndrome was diagnosed. The patient was treated with bortezomib, dexamethasone, and lenalidomide. After four treatment cycles, the patient’s symptoms showed significant improvement (Figures 1e,f; Table 1).

**Table 1:** Comparison of key clinical and laboratory parameters before and after treatment.

Evaluation index	Baseline (before treatment)	After treatment (4 cycles)	Reference scope
Main symptoms	Severe abdominal distension, bone pain, itching and numbness in the lower limbs	Significantly alleviated	–
VEGF (pg/mL)	209.88 (Increase)	75.52 (Normal)	<160.00
Platelet count (×10^9^/L)	115 × 10^9^/L (Decline)	81 × 10^9^/L (Decline)	125–350 × 10^9^/L
Thyroid function (TSH)	5.96 uIU/mL (Increase)	1.69 uIU/mL (Normal)	0.27–4.2 uIU/mL
Ascit	Large in quantity and stubborn	Small amount	–
Size of the spleen	Splenomegaly	Mild splenomegaly (smaller than before)	–

## Discussion

POEMS syndrome is a rare cause of ascites. It is often missed or misdiagnosed and has a mortality rate as high as 39% (5). This case reminds us that in patients with refractory peritoneal effusion of unknown cause who also present peripheral neuropathy, organ enlargement, or endocrine disorders, screening VEGF levels and M protein is necessary to improve the diagnosis and prognosis of POEMS syndrome, a rare cause of ascites.

Ascites is the pathological accumulation of fluid in the abdominal cavity and a common clinical sign of various diseases. Its etiology is complex, and accurate identification is crucial for guiding treatment. Common causes of ascites include liver cirrhosis (27.9% of adult cases) (6), malignant tumors such as metastatic ovarian or gastrointestinal cancer (28.9%) (7), heart failure, nephrotic syndrome, tuberculous peritonitis, and pancreatitis (6, 8, 9). The nature of ascites closely relates to its etiology, and clinicians classify it mainly into transudates and exudates based on indicators such as appearance, protein content, cell count, and bacterial culture. Transudates are usually clear and have low protein content. They are commonly seen in liver cirrhosis or heart failure. Exudates, on the other hand, are turbid with high protein content and are often found in malignant tumors or infections. However, the traditional method of classifying ascites based on nature (such as transudates and exudates) has low accuracy. It has since been replaced by a pathophysiological classification using the serum-ascites albumin gradient (SAAG) as the gold standard. A SAAG ≥ 1.1 g/dL (high gradient) indicates portal hypertension-related ascites, such as cirrhotic or cardiogenic ascites. This classification has a diagnostic accuracy exceeding 97%. Conversely, a SAAG < 1.1 g/dL (low gradient) suggests non-portal hypertension ascites, including malignant ascites, tuberculous peritonitis, or pancreatogenic ascites (10).

In the case we reported, the molecular and cytopathological report of ascites indicated no tumor cells or *Mycobacterium tuberculosis*. The routine and biochemical reports of ascites suggested a transudate. Considering the results of routine blood tests, chest and abdominal CT, and the poor response to diuretics and catheter drainage of ascites, common causes such as liver cirrhosis, malignant tumors, heart failure, nephrotic syndrome, and tuberculous peritonitis were unlikely. Therefore, rare causes of ascites needed to be explored.

POEMS syndrome is a rare plasma cell neoplasm associated with paraneoplastic syndrome (11). Its latest diagnostic criteria require the simultaneous satisfaction of: two mandatory criteria (polyneuropathy and monoclonal plasma cell abnormality), at least one major criterion (osteosclerotic lesions/Castleman disease/elevated VEGF), and at least one minor criterion (such as thrombocytosis, endocrine disorders, organomegaly, ascites, etc.) (12). This case meets two mandatory criteria, two major criteria (osteosclerotic lesions + elevated VEGF), and multiple minor criteria (endocrine disorders + organomegaly + ascites); therefore, the diagnosis of POEMS syndrome is clear. In terms of the mandatory criteria, Although multiple neuropathy is the core feature and essential diagnostic criterion of POEMS syndrome, an increasing amount of evidence indicates the existence of an extremely rare atypical variant that lacks obvious neuropathy (13, 14). The diagnosis of such cases is highly challenging and mainly relies on the identification of other key features such as vascular endothelial growth factor (VEGF) levels, sclerotic bone lesions, and Castleman disease (15). Although there are no officially revised diagnostic criteria to cover this subgroup at present, clinicians should remain highly vigilant about it. For patients who exhibit other systemic characteristics of POEMS syndrome, even if they have no neurological symptoms, a comprehensive assessment should be conducted to avoid missed diagnosis and mistreatment. An in-depth study of this rare variant will help broaden our understanding of POEMS syndrome, a complex disease. In terms of the major criteria, according to the literature review, “severe bone pain” is not the official diagnostic criterion for POEMS syndrome (16). However, in the course of this patient’s disease, after seeking medical attention due to bone pain, imaging examinations revealed bone changes. Eventually, combined with the patient’s systemic multi-system manifestations and related tests, the diagnosis was POEMS syndrome. This suggests that although bone pain itself is not an official diagnostic criterion, it is an important clinical signal indicating the need for imaging examinations to detect bone lesions that are the main diagnostic criteria for POEMS syndrome. Literature indicates that in POEMS syndrome, bone lesions are extremely common, with approximately 95% of patients having bone changes, mainly manifested as sclerotic or mixed (coexistence of sclerotic and osteolytic) lesions, usually involving axial bones such as the pelvis, spine and ribs, while simple sclerotic lesions mostly have no obvious symptoms (17). The patient’s severe bone pain mainly stems from the osteolytic components in the lesion rather than bone sclerosis itself. This is because osteolytic lesions are accompanied by more active metabolism, plasma cell infiltration and possible microstructure destruction, which stimulate nerve endings and cause pain. Therefore, when patients with POEMS syndrome complain of severe bone pain, clinicians should highly suspect that there may be osteolytic or active mixed components in their bone lesions, rather than just stable, purely sclerotic lesions (18). In addition, since no significant enlargement of superficial or deep lymph nodes was found in the physical examination and imaging examination of this case, lymph node biopsy was not performed. According to the literature review, although POEMS syndrome and Castleman disease are independent diseases, they often overlap in clinical practice. Approximately 11–30% of POEMS patients also have multicenter Castleman disease. In addition, Castleman disease can be used as one of the diagnostic criteria for POEMS (19, 20). There is also an intersection in pathology between the two. POEMS places more emphasis on damage to multiple systems, including the nervous, endocrine and skin aspects, while Castleman’s disease centers on lymph node lesions. When these two diseases are associated, the complexity of diagnosis increases. Therefore, a comprehensive judgment needs to be made through pathological biopsy, M protein detection and VEGF level analysis. In terms of the minor criteria, according to the literature, thrombocythemia is a hematological abnormality with an extremely high incidence rate among patients with POEMS syndrome and is an important component of this disease. However, in approximately 10–20% of cases, platelets may be normal or even reduced (21). This case report did not show typical thrombocythemia. During the subsequent treatment process, the dynamic platelet count monitoring data mostly decreased, which may be related to individual differences or concurrent splenomegaly and other factors. Nevertheless, given that POEMS syndrome itself is regarded as a hypercoagulable state, even if platelets are normal, the risk of thrombosis still needs to be vigilant (22). In addition, The vast majority of the literature clearly indicates that ascites related to POEMS syndrome is of the exudate nature (23). However, in this case, the ascites routine and biochemical reports of the patient suggest a tendency of exudate (Rivalta test weakly positive, total protein 37.7 g/L). This difference may be due to the following reasons. Firstly, in this case, the patient simultaneously had renal insufficiency and hypoproteinemia, which led to the ascites protein content possibly falling precisely within the range of the exudate. The second is the limitation of the diagnostic criteria themselves. The traditional classification methods of ascites nature (such as exudate and leakage fluid) have relatively low accuracy. At this time, more attention should be paid to the low serum-ascites albumin gradient (SAAG)‍, which is the core indicator for excluding portal hypertension. Several studies have shown that ascites in POEMS usually presents as low-gradient ascites (SAAG < 1.1 g/dL), which clearly points to the fact that its cause is not portal hypertension (24). Although SAAG was not detected in this case, considering its refractory characteristics and multi-system manifestations throughout the body, the diagnosis of POEMS is still strongly supported. Therefore, when encountering low SAAG ascites of unknown cause, even if it is reported as “exudative,” POEMS syndrome still needs to be included in the differential diagnosis and a comprehensive assessment should be conducted. Moreover, according to the literature review, in POEMS syndrome, abnormally elevated vascular endothelial growth factor (VEGF) is considered the core factor in the formation of ascites (25). VEGF activates multiple downstream signaling pathways, including Src, PI3K/AKT and MAPK, by binding to its major receptor VEGFR-2 (26). The activation of these signaling pathways leads to the dysfunction of endothelial intercellular junction proteins, especially the down-regulation of protein expressions such as VE-cadherin in adhesion junctions and claudin-5 and ZO-1 in tight junctions (27, 28). This series of molecular events disrupted the integrity of the vascular endothelial barrier, leading to a significant increase in vascular permeability. As a result, plasma components (including fluids and proteins) seeped into the interstitial Spaces and serous cavities (such as peritoneal and pleural cavities), ultimately causing ascites, pleural effusion and systemic edema (29). Although its mechanism has been clarified, there is still a lack of clinical cohort studies that directly quantify the correlation between VEGF concentration and the severity of ascites (such as ascites volume and serum-ascites albumin gradient SAAG).

Regarding treatment, POEMS ascites is often resistant to diuretics; therefore, therapy targeting the primary disease, such as controlling plasma cell clones and inhibiting VEGF, is required. Multiple studies have consistently shown that autologous stem cell transplantation (ASCT) is currently the most effective treatment for POEMS syndrome, which can significantly improve the long-term prognosis of patients, with a 5-year survival rate exceeding 90%. In addition, this therapy can also effectively alleviate core symptoms such as neuropathy (30). ASCT is suitable for patients who are usually under 65 years old and have good organ function. Although there are certain treatment risks, under strict screening and management conditions, the benefits it brings far outweigh the risks, and it has become the first-line core treatment option for this disease (31). Since the patient in this case is a 70-year-old elderly male with complications such as renal insufficiency, autologous stem cell transplantation (ASCT) was not adopted; instead, a chemotherapy regimen of bortezomib + dexamethasone + lenalidomide was used, and favorable therapeutic effects were achieved.

## Conclusion

POEMS syndrome presenting with refractory peritoneal effusion as the main or initial symptom is prone to missed or incorrect diagnosis, with mortality rates reaching 39% (5). This case highlights that patients with refractory peritoneal effusion of unknown cause should be evaluated for accompanying symptoms such as peripheral neuropathy, organ enlargement, and endocrine disorders. In such cases, screening for VEGF levels and M protein is essential. Moreover, by analyzing the mechanisms and applying targeted therapies to overcome current treatment limitations, we can improve the diagnosis and prognosis of POEMS syndrome, a rare cause of ascites.

## Data Availability

The raw data supporting the conclusions of this article will be made available by the authors, without undue reservation.
